# Anti-retroviral therapy after “Treat All” in Harare, Zimbabwe: What are the changes in uptake, time to initiation and retention?

**DOI:** 10.12688/f1000research.23417.2

**Published:** 2020-08-20

**Authors:** Takura Matare, Hemant Deepak Shewade, Ronald T. Ncube, Kudzai Masunda, Innocent Mukeredzi, Kudakwashe C. Takarinda, Janet Dzangare, Gloria Gonese, Bekezela B. Khabo, Regis C. Choto, Tsitsi Apollo

**Affiliations:** 1 AIDS and TB Unit, Ministry of Health and Child Care, Harare, Zimbabwe; 2 The Union South-East Asia Office, New Delhi, India; 3 International Union Against Tuberculosis and Lung Disease (The Union), Paris, France; 4 The Union Zimbabwe Office, Harare, Zimbabwe; 5 Harare City Health Department, Harare, Zimbabwe; 6 International Training and Education Centre for Health (I-TECH), Harare, Zimbabwe

**Keywords:** ART outcomes, test and treat, universal test and treat, time to treatment, HIV, SORT IT, Operational research

## Abstract

**Background: **In Zimbabwe, Harare was the first province to implement “Treat All” for people living with human immunodeficiency virus (PLHIV). Since its roll out in July 2016, no study has been conducted to assess the changes in key programme indicators. We compared antiretroviral therapy (ART) uptake, time to ART initiation from diagnosis, and retention before and during “Treat All”.

**Methods: **We conducted an ecological study to assess ART uptake among all PLHIV newly diagnosed before and during “Treat All”. We conducted a cohort study to assess time to ART initiation and retention in care among all PLHIV newly initiated on ART from all electronic patient management system-supported sites (n=50) before and during “Treat All”.

**Results: **ART uptake increased from 65% (n=4619) by the end of quarter one, 2014 to 85% (n=5152) by the end of quarter four, 2018.  A cohort of 2289 PLHIV was newly initiated on ART before (April-June 2015) and 1682 during “Treat all” (April-June 2017). Their age and gender distribution was similar. The proportion of PLHIV in early stages of disease was significantly higher during “Treat all” (73.2% vs. 55.6%, p<0.001). The median time to ART initiation was significantly lower during “Treat All” (31 vs. 88 days, p<0.001). Cumulative retention at three, six and 12 months was consistently lower during “Treat all” and was significant at six months (74.9% vs.78.1% p=0.022).

**Conclusion: **Although there were benefits of early ART initiation during “Treat All”, the programme should consider strategies to improve retention.

## Introduction

Globally in 2018, there were an estimated 37.9 million people living with human immunodeficiency virus (PLHIV) ^1^. The majority of them (54%) are in eastern and southern Africa. Since the start of the epidemic, 32 million people were estimated to have died from acquired immune deficiency syndrome related illnesses ^1^. An estimated 23 million people are accessing antiretroviral therapy (ART) globally ^1^.

Following evidence from research studies on the clinical and public health benefits of immediate ART, in July 2015, the World Health Organization (WHO) released guidelines on when to start ART and pre-exposure prophylaxis for HIV ^2, 3^. These guidelines recommended ART to be offered to all PLHIV (“Treat All”), regardless of CD4 threshold and/or WHO clinical stage. In July 2018, WHO reported that 84% of low- and middle-income countries had adopted the “Treat All” policy ^4^. Studies from southern African countries have demonstrated the health and economic benefits of “Treat All” ^5^. Research in rural South Africa and Malawi demonstrated that after implementation of “Treat All”, people on ART had better retention in care ^6, 7^.

Zimbabwe has a generalized HIV epidemic, with an estimated 1.3 million PLHIV and an HIV prevalence of 14% among adults (15–49 years) as per the ZIMPHIA survey 2015–16 ^8, 9^. In July 2016, Harare province was the first to start implementing “Treat All” ^10^. Since the roll out of “Treat All”, no comparative study has been conducted to measure the changes in linkage to care and ART outcomes. We therefore assessed ART uptake, time to ART initiation from diagnosis and retention among PLHIV before and during “Treat All” in Harare.

## Methods

### Study design and population

For ART uptake, we used an ecological design involving aggregate secondary programme data and all PLHIV newly diagnosed before (2014 to June 2016) and during “Treat All” (July 2016 to 2018) in Harare were the study population.

For time to ART initiation and retention, we used a cohort design involving patient wise secondary programme data. We included all PLHIV newly initiated on ART from 50 electronic patient management system (ePMS) sites in Harare before (April–June 2015) and during “Treat All” (April–June 2017).

### Study setting

***General setting***. Harare province constitutes 16.3% of the population in Zimbabwe ^11^, with an estimated 0.2 million PLHIV, the highest among all the provinces ^8^. It has the highest number of patients active on ART, with 77 public ART sites providing HIV diagnosis and treatment. As on October 2018, the 50 ePMS sites constituted 52% of all people on ART in Harare province.

***ART initiation before “Treat All”***. Prior to “Treat All”, PLHIV were initiated on ART based on a CD4 eligibility criteria of <500 cells/mm ^3^ (with priority given to those <300 cells/mm ^3^ OR WHO clinical stage 3 or 4). Additionally, pregnant and/or breast feeding women, sero-discordant couples, Hepatitis B virus co-infection, people diagnosed with tuberculosis (TB) and children ≤5 years were also eligible irrespective of CD4 count.

Baseline investigations at primary level of care included urine dipstick (glucose, protein), haemoglobin, CD4 count for immunologic staging, cryptococcal antigen screen for adults with CD4 count <100 cells/mm ^3^, screen for pregnancy, syphilis if 12 years or older and hepatitis B infection ^12^. Other investigations included full blood count, creatinine and hepatitis C serology at secondary or tertiary levels of care. PLHIV were screened for active TB and other Opportunistic Infections (OIs) ^12^. If active TB was diagnosed, ART was initiated within 2–8 weeks of initiation of anti-TB treatment and within two weeks for advanced TB disease.

Psychosocial criteria for ART initiation, also included completion of prescribed counselling sessions and an assessment of adherence to cotrimoxazole preventive therapy (CPT) in the past 3 months. Adherence to CPT was used to assess the likelihood that the patient would adhere to ART. Patients were followed up monthly initially and then every three months. Viral load testing targeted those suspected of HIV treatment failure ^13^. A patient with confirmed virologic, immunologic or clinical failure were switched to second line ART ^12^. Data were routinely recorded in the ART register, patient OI/ART care booklet. The patient level data was also routinely captured electronically in the 50 ePMS sites.

If a patient officially transfers-out to another ART clinic, they are issued with a transfer-out letter for presentation at the receiving ART clinic and they maintain their ART number. In other instances, patients may unofficially transfer out to another health facility without being assigned a transfer-out letter and are therefore declared lost to follow-up if they exceed 90 days after their last scheduled clinic or drug pick-up visit and all phone-call or physical follow-ups attempts by community health workers to trace them back to care have been futile. The patients will often present at the transfer-in facility with a patient-held ART booklet which indicates their previous medical history and therefore continue receiving ART care whilst also maintaining their ART number. Alternatively, if no evidence of previous treatment is provided, they are offered an HIV test and subsequently assigned a new ART number and treated as new ART client

***Changes in ART initiation and further management during “Treat All”***. All PLHIV are eligible for ART regardless of CD4 count or WHO clinical staging ^10^ although baseline CD4 monitoring is still important for determining the degree of immune suppression of a patient in order to inform ‘differentiated care’. Additional changes in the “Treat All” period included an emphasis for health workers to provide adequate counselling and start ART within a week with exception of pregnant and breast-feeding women who were to be started on ART on the same day of HIV diagnosis. However, for those patients who are not ready yet to start ART, they should receive on-going counselling and support. PLHIV are monitored for viral load at six months after starting ART, 12 months then annually thereafter if stable ^12^.

### Data variables and sources of data

For ART uptake, we extracted quarterly aggregate number of new HIV diagnoses and new ART initiations from the District Health Information System 2 (DHIS 2). For time to ART initiation and retention, the source of patient level data was the ePMS and included OI/ART number; ART site; date of HIV diagnosis; date of ART initiation; date of birth; baseline characteristics and outcome at three, six and 12 months - alive and on treatment, death, loss to follow up, stopped ART, transferred out; and date of ART outcome. Operational definition of outcomes has been depicted in Table 1.

**Table 1.** Operational definitions for ART outcomes for PLHIV, Zimbabwe (2015–19).

Outcome	Operational Definition
Alive and on treatment	Adults and children with HIV known to be on treatment
Transferred out	Documented transfer status in the ePMS/individual ART booklet
Died	Documented with a death outcome in the ePMS/individual ART booklet
Lost to follow up	Patients who have not attended their last scheduled review visit or pill pick-up visit by more than 90 days from the date of data collection.
Stopped treatment	Documented as having stopped ART in the individual ART booklet, pre- ART or ART registers
Attrition	Death, loss to follow up and stopped treatment combined
Retained in care	Alive and on treatment and transferred out combined. Patients that are transferred out were censored on their last recorded visit to ART site

HIV: human immunodeficiency virus; PLHIV: people living with HIV; ART: antiretroviral therapy; ePMS: electronic patient management system.

### Analysis and statistics

For ART uptake, we calculated quarterly proportions of newly diagnosed PLHIV initiated on ART (Microsoft Excel 2010, Microsoft, Redmond, WA, USA) and presented them using bar diagram and trend lines.

We summarized time to ART initiation after diagnosis using median (interquartile range - IQR) and compared it before and during “Treat All” using Mann Whitney U test. We also used frequency and proportions to summarize time to ART (same day, 2–7 days, 8–14, 15–30 days, 31–90 days, 91–180 days, >180 days] and compared it before and during “Treat All” using z test for proportions. Baseline characteristics and cumulative retention in care were also compared using z test for proportions (STATA - version 12.1, copyright 1985–2011 Stata Corp LP USA).

### Ethics

We obtained ethics approval from the Union Ethics Advisory Group (EAG), Paris, France (EAG number: 47/19, 29 April 2019) and the Medical Research Council of Zimbabwe (MRCZ), Harare (MRCZ/E/245, 19 August 2019). As this study involved review of existing programme records, we sought and obtained waiver of written informed consent from the ethics committees. Secondary data were extracted after obtaining administrative approval from the concerned authorities.

## Results

### ART uptake

**Figure 1.**
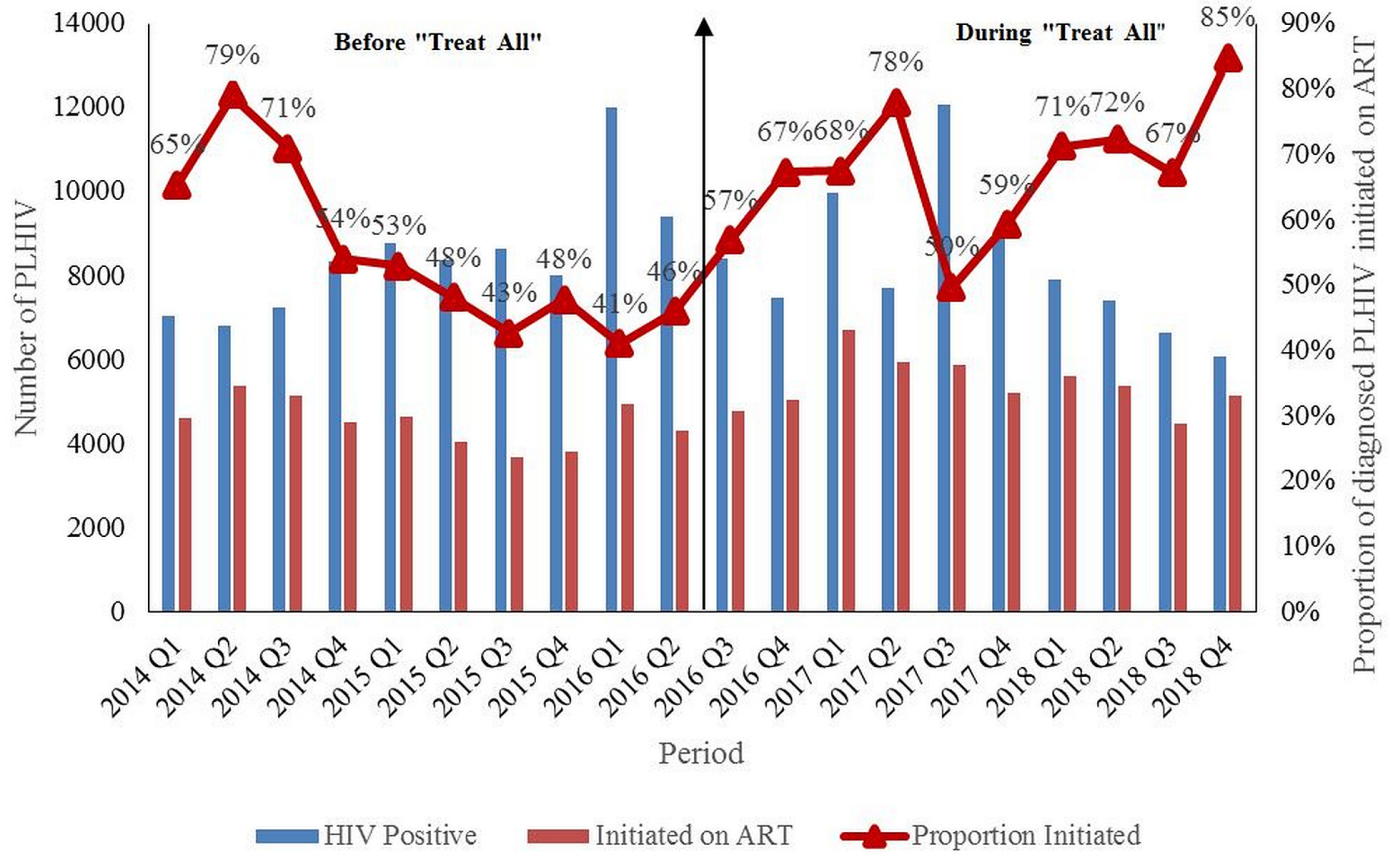
Trends ^#^ in quarterly ART uptake^ among newly diagnosed PLHIV before (January 2014–June 2016) and during (July 2016–December 2018) “Treat All” *, Harare, Zimbabwe. PLHIV: People Living with Human Immunodeficiency Virus; ART: Antiretroviral therapy ^aggregate numbers for each quarter were extracted to calculate ART uptake, source of data is district health information system (DHIS-2) *” Treat All” means all individuals with confirmed HIV diagnosis are eligible for ART irrespective of WHO clinical stage or CD4 count. ^#^During 2014, the CD4 count eligibility criteria was raised from <350 to <500 cells/mm ^3^ ^3^. As more people in pre-ART care were eligible for ART, there was an increase in new ART initiations which resulted in corresponding increase in ART uptake. Similarly, there was an increase in new ART initiations which resulted in corresponding increase in ART uptake after “Treat All”.

A total of 84,776 people were diagnosed with HIV before (2014 to June 2016) and 82,672 during “Treat All” (July 2016 to 2018). ART uptake increased from 65% (n=4619) by the end of quarter one, 2014 to 85% (n=5152) by the end of quarter four, 2018. Starting quarter three, 2016 (implementation of “Treat All”), there was an increasing trend in the new ART initiations, as well as the ART uptake up to quarter two, 2017. Between quarter three, 2017 and quarter four, 2018, there was an increasing trend in ART uptake, which peaked to 85% (n=5152) ( Figure 1). However, during this period, the absolute number of HIV diagnoses as well as the absolute new ART initiations reduced.

### Baseline characteristics among PLHIV newly initiated on ART

A total of 2289 PLHIV were newly initiated on ART before (April-June 2015) and 1682 were newly initiated during “Treat all” (April-June 2017). The median age (in years) for the two groups was similar, 31 (IQR: 27, 41). Gender distribution before and during “Treat all” was also similar (women: 58.6% vs. 58.3% respectively). The proportion of people in early stages of disease (WHO clinical stage I or II) was significantly higher during “Treat all” (73.2% vs. 55.6%, p<0.001).

### Time to ART initiation from diagnosis

We were not able to calculate the time interval for 41% of people because of missing dates. Of those with dates available, the median time to ART initiation from diagnosis was significantly lower during “Treat All” when compared to before “Treat all” (31 vs. 88 days, p<0.001). ART initiation on the same day (19.3% vs. 4.9%, p=<0.001) and within 14 days (25.5% vs 12.5%, p<0.001) was significantly higher during “Treat All” ( Table 2).

**Table 2.** Time to ART initiation from diagnosis among PLHIV newly initiated on ART, before (April–June 2015) and during “Treat All” * (April–June 2017) in 50 ePMS ART sites ^ in Harare, Zimbabwe.

Time to ART initiation (in days)	Before “Treat All”		During “Treat All”	
	N	(%)	N	(%)
**Total**	**2289**	**(100.0)**	**1682**	**(100.0)**
Same day	112	(4.9)	325	(19.3)
2–7	69	(3.0)	57	(3.5)
8–14	106	(4.6)	45	(2.7)
15–30	121	(5.3)	59	(3.5)
31–90	273	(11.9)	179	(10.6)
91–180	292	(12.8)	239	(14.2)
>180	369	(16.1)	88	(5.2)
Not recorded	947	(41.4)	690	(41.0)

PLHIV: people living with human immunodeficiency virus; ART: antiretroviral therapy; ePMS: electronic patient management system. *”Treat All” means all individuals with confirmed HIV diagnosis are eligible for ART irrespective of WHO clinical stage or CD4 count. ^As on October 2018, the 50 ePMS sites constituted 52% of all people on ART in Harare province.

### ART retention

Retention at three, six and 12 months was consistently lower during “Treat All” compared to before “Treat All”. It was significantly lower at six months (74.9% vs.78.1% p=0.022) ( Table 3).

**Table 3.** Retention at three, six, and 12 months among PLHIV newly initiated on ART, before (April–June 2015) and during “Treat All” * (April–June 2017) in 50 ePMS ART sites ^ in Harare, Zimbabwe.

ART outcomes	Before “Treat All”		During “Treat All”		P – value ^#^
	N	%	N	(%)	
**Total**	**2289**	**(100.0)**	**1682**	**(100.0)**	
**Three months**					
*Retained in care*	1905	(83.2)	1359	(80.8)	0.053
*Attrition*					
Died	2	(<0.1)	0	(0.0)	
LTFU	381	(16.7)	320	(19.0)	
Stopped ART	1	(<0.1)	3	(<0.1)	
**Six months**					
*Retained in care*	1787	(78.1)	1260	(74.9)	**0.02**
*Attrition*					
Died	2	(<0.1)	0	(0.0)	
LTFU	499	(21.8)	419	(24.9)	
Stopped ART	1	(<0.1)	3	(<0.1)	
**12 months**					
*Retained in care*	1667	(72.8)	1203	(71.5)	0.38
*Attrition*					
Died	2	(<0.1)	1	(<0.1)	
LTFU	619	(27.1)	475	(28.3)	
Stopped ART	1	(<0.1)	3	(<0.1)	

PLHIV: people living with human immunodeficiency virus; ART: antiretroviral therapy; ePMS: electronic patient management system; LFTU: loss to follow up. *” Treat All” means all individuals with confirmed HIV diagnosis are eligible for ART irrespective of WHO clinical stage or CD4 count. ^As on October 2018, the 50 ePMS sites constituted 52% of all people on ART in Harare province. ^#^z test of proportions (proportion retained in care).

## Discussion

This is the first study in Zimbabwe that attempts to document the changes in ART initiation and retention before and during “Treat All”. There were three programme relevant findings.

First, ART uptake improved immediately after the implementation of “Treat All” (starting July 2016 ^10^). When compared to quarter one, 2014 (65%), ART uptake improved in quarter four, 2018 (85%). However, there was a drop in the number of PLHIV diagnosed and number of new ART initiations from quarter three, 2017 to quarter four, 2018. The improvement in uptake during this period was not due to increase in annual new ART initiations. It was because of faster falling trends of HIV diagnoses relative to the falling trends of new ART initiations. Hence, the programme should explore the reasons for reduction in new ART initiations and take necessary action to ensure that it moves closer to the second ‘90’ of UNAIDS 90-90-90 targets by 2020 ^14^.

Second, time to ART initiation significantly reduced during “Treat All” when compared to before “Treat All” (31 vs. 88 days). Though the median time reduced, the proportion initiated on ART on the day of diagnosis was less compared to 65% in mission hospitals of Zimbabwe in 2017 ^15^. Therefore there is further opportunity to reduce time to linkage to ART. Faster linkage is known to be associated with better retention ^16^.

Third, retention did not improve despite more PLHIV being clinically asymptomatic at ART initiation during “Treat All” when compared to before “Treat All”. This was contrary to findings in Malawi, where retention at 12 months during “Treat All” (83%) was higher than before “Treat All” (76%) ^7^. In Kenya and Uganda (2017), retention at one year was 89% ^16^. In mission hospitals of Zimbabwe (2017), retention at three months was as high as 90% ^15^. Good health (at diagnosis) has been reported to act both as a barrier as well as a facilitator to ART initiation ^17, 18^, therefore, we speculate that good health at ART initiation may also act both as a barrier as well as a facilitator to ART retention. Another potential reason for the observed lower retention could be deficiencies in coverage of quality adherence counselling with likely increased work load during the “Treat All” era. High rates of LTFU before and during “Treat All” may not actually be LTFU but ‘silent transfers’ where patients enrol in a different clinic closer to home without being issued an official transfer-out letter. Due to stigma and discrimination, people tend to get tested out of their area of residence where they are not known and after enrolment tend to move closer to home for treatment.

The programme may consider combination intervention strategy to improve linkages to care and retention. This strategy includes i) point-of-care CD4 testing at the time of diagnosis, ii) accelerated ART initiation, and iii) short message service (SMS) health messages and appointment reminder ^19^. The programme should consider qualitative systematic enquiry into why the retention did not improve during “Treat All” in Harare.

The strength of this study were the large sample size, which included all sites in Harare reporting through DHIS 2 and ePMS. There were however some limitations. First, inherent to observational studies is the possibility of documentation errors that could not be validated. Second, some baseline characteristics namely; body mass index, CD4 count, anaemia and Hepatitis B and C co-infections were incomplete in at least 80% of the records and were excluded from analysis. PLHIV transferred out were censored on their date of transfer out as we did not have details about their registration into the ‘transfer in’ ART clinic.

In conclusion, as expected, “Treat All” increased ART uptake and reduced time to ART initiation. Retention in care did not improve as a result of “Treat all”. This is a clarion call for the programme to focus interventions on efficient linkage to ART and retention in care in order to reap the benefits of “Treat All”.

## Data availability

### Underlying data

Figshare: Matare T et study 2020 ART uptake, initiation delay and retention data, https://doi.org/10.6084/m9.figshare.c.4944399 ^20^.

This project contains the following underlying data:

- *ART initiation delay and retention data:* Patient wise ART initiation delay and retention data of people initiated on ART before (Q2-2015) and during (Q2-2017) "Treat All" from the 50 ePMS sites in Harare, Zimbabwe.
- *ART uptake data:* Aggregate quarterwise ART uptake data from 2014 to 2018 in Harare.

Data are available under the terms of the Creative Commons Zero "No rights reserved" data waiver (CC0 1.0 Public domain dedication).
