# Supplementary material for: Anti-retroviral therapy after “Treat All” in Harare, Zimbabwe: What are the changes in uptake, time to initiation and retention?
Source: F1000Res. 2020 Aug 20;9:287. Originally published 2020 Apr 24. [Version 2] doi: 10.12688/f1000research.23417.2 (PMC7475956; doi:10.12688/f1000research.23417.2)
Supplement: Peer review history

# Peer review: Reviewer response for version 2

**DOI:** 10.5256/f1000research.28423.r70220

**Published:** 2020-09-17

**Authors:** Aneesa Moolla, Nalini Naidoo

**Affiliations:** 1 Department of Internal Medicine, Health Economics and Epidemiology Research Office, School of Clinical Medicine, Faculty of Health Sciences, University of the Witwatersrand, Johannesburg, South Africa; 2 Department of Internal Medicine, Health Economics and Epidemiology Research Office, School of Clinical Medicine, University of the Witwatersrand, Johannesburg, South Africa

Thank you for allowing us the opportunity to review this manuscript that addresses a pertinent issue in many parts of Africa.

 Literature and Rationale:

- Actual long-term reasons for conducting such a study needs to be explained more clearly here in the literature section which ideally should end with a rationale for the study.

Study Design and Population:

- At what level of care were the 50 ePMS sites?
- What does "patient wise secondary programme data" mean? Please explain.

Changes in ART initiation and further management during “Treat All”:

- What was the frequency and please explain on-going counselling and support for those patients who are not ready yet to start ART.   

Analysis and statistics:

- You draw somewhat ambitious conclusions relative to these tests you run here...which are simply hypothesis tests. Please confirm that these tests as the best you could have run for the conclusions you draw. A statistician consultant confirmation can be mentioned.

Results:

 ART Uptake

- Results are confusing, before ‘Treat All’ what % of the 84776 people diagnosed with HIV qualified for ART? Is the 65% ART uptake (n=4619) related to this cohort? If so, ART uptake should be 55104.
- Same comment for ‘Treat All’.
- May be worth moving ‘Baseline characteristics among PLHIV….ART’ to above ‘ART Uptake’ as some of these issues are resolved in the ‘Baseline characteristics’ section.

Is the work clearly and accurately presented and does it cite the current literature?

Yes

If applicable, is the statistical analysis and its interpretation appropriate?

Partly

Are all the source data underlying the results available to ensure full reproducibility?

Yes

Is the study design appropriate and is the work technically sound?

Yes

Are the conclusions drawn adequately supported by the results?

Yes

Are sufficient details of methods and analysis provided to allow replication by others?

Yes

Reviewer Expertise:

Public health with a focus on the HIV care cascade, early childhood development, TB care and treatment and adolescent preferences for healthcare services.

We confirm that we have read this submission and believe that we have an appropriate level of expertise to confirm that it is of an acceptable scientific standard, however we have significant reservations, as outlined above.

# Peer review: Reviewer response for version 2

**DOI:** 10.5256/f1000research.28423.r69887

**Published:** 2020-09-04

**Authors:** Boniface Dongmo‐Nguimfack

**Affiliations:** 1 Department of HIV/AIDS, World Health Organization, Geneva, Switzerland

All corrections requested have been taken into consideration and dealt with. I am satisfied with the tone of the paper and the quality of the analysis. This paper is good for indexing.

Is the work clearly and accurately presented and does it cite the current literature?

Partly

If applicable, is the statistical analysis and its interpretation appropriate?

Yes

Are all the source data underlying the results available to ensure full reproducibility?

Yes

Is the study design appropriate and is the work technically sound?

Partly

Are the conclusions drawn adequately supported by the results?

Yes

Are sufficient details of methods and analysis provided to allow replication by others?

Yes

Reviewer Expertise:

I am an economist and member of the HIV guideline Technical Working Group of the WHO

I confirm that I have read this submission and believe that I have an appropriate level of expertise to confirm that it is of an acceptable scientific standard.

# Response

**Published:** 2020-09-04

**Authors:** Hemant Deepak Shewade

**Affiliations:** International Union Against Tuberculosis and Lung Disease, India

Thank you for the constructive feedback. We are happy that the manuscript now meets your expectations.

# Peer review: Reviewer response for version 2

**DOI:** 10.5256/f1000research.28423.r69888

**Published:** 2020-09-04

**Authors:** Joseph Mugisha Okello

**Affiliations:** 1 Uganda Research Unit, Medical Research Council/Uganda Virus Research Institute and London School of Hygiene and Tropical Medicine, Entebbe, Uganda

I am happy with the responses from the authors.

Is the work clearly and accurately presented and does it cite the current literature?

Partly

If applicable, is the statistical analysis and its interpretation appropriate?

Yes

Are all the source data underlying the results available to ensure full reproducibility?

Yes

Is the study design appropriate and is the work technically sound?

Yes

Are the conclusions drawn adequately supported by the results?

Yes

Are sufficient details of methods and analysis provided to allow replication by others?

Partly

Reviewer Expertise:

NA

I confirm that I have read this submission and believe that I have an appropriate level of expertise to confirm that it is of an acceptable scientific standard.

# Peer review: Reviewer response for version 1

**DOI:** 10.5256/f1000research.25846.r64871

**Published:** 2020-07-16

**Authors:** Joseph Mugisha Okello

**Affiliations:** 1 Uganda Research Unit, Medical Research Council/Uganda Virus Research Institute and London School of Hygiene and Tropical Medicine, Entebbe, Uganda

Thank you very much for giving me the opportunity to review this manuscript. I hope my review comments will lead to further improvement of the manuscript.

**General comments:**

1. The manuscript addresses important research questions about changes of uptake, time to initiation and retention.
2. Adherence to ART is an important issue in the management of patients in ART programmes. I am surprised that the authors did not look at adherence while looking at key programme indicators. Although the authors indicate how monitoring of patients using viral loads was done before and after treat all, they did not look at this as one of their study outcomes. The authors need to acknowledge this as one of the weaknesses of this study.

 

**Specific comments:**

**Introduction:**

1. In paragraph 2 of the introduction, the authors say that research in other countries demonstrated that after implementation of the “treat all”, people on ART had better outcomes. The authors should mention these countries and specifically mention which ART outcomes were better.
2. The authors need to give more details on the 14% prevalence of HIV in Zimbabwe. The references given are for 2012, 2016 and 2018. Is the current HIV prevalence in Zimbabwe still 14%? How was this national prevalence of HIV estimated in Zimbabwe?
3. In the introduction, the authors should mention the proportion of HIV infected people who were taking ART before the treat all policy became operational and the proportion currently when treat all policy is operational.

 

**Methods:**

1. Can you give more information on the 77 ART sites providing HIV diagnosis and treatment? Are these public facilities or private facilities?
2. Are there private providers of ART in Harare? If they are there, is the information captured by District health Information systems?
3. Is data on ART adherence captured at these ART treatment sites?
4. When people on ART get lost to follow up, is there anything done to establish reasons why they got lost to follow up?

** **


**Discussion:**

1. You need to acknowledge the weakness of not looking at adherence in this study. You actually seem to agree with me on this point where you say “another potential reason for the observed lower retention could be deficiencies in coverage of quality adherence counselling with likely increased work load during the “treat all era”.
2. You also need to recommend qualitative studies to look at why retention remained low in the treat all era in Harare compared to other studies in other countries. You rightly said you speculated. 

Is the work clearly and accurately presented and does it cite the current literature?

Partly

If applicable, is the statistical analysis and its interpretation appropriate?

Yes

Are all the source data underlying the results available to ensure full reproducibility?

Yes

Is the study design appropriate and is the work technically sound?

Yes

Are the conclusions drawn adequately supported by the results?

Yes

Are sufficient details of methods and analysis provided to allow replication by others?

Partly

Reviewer Expertise:

epidemiology, HIV and ageing, chronic conditions

I confirm that I have read this submission and believe that I have an appropriate level of expertise to confirm that it is of an acceptable scientific standard, however I have significant reservations, as outlined above.

# Response

**Published:** 2020-07-28

**Authors:** Hemant Deepak Shewade

**Affiliations:** International Union Against Tuberculosis and Lung Disease, India

Thank you very much for the invaluable comments. We respond below, point by point.

**Reviewer:**

Thank you very much for giving me the opportunity to review this manuscript. I hope my review comments will lead to further improvement of the manuscript.

**Response:**

Thank you very much.

**Reviewer:**

General comments:

The manuscript addresses important research questions about changes of uptake, time to initiation and retention.

**Response:**

Thank you very much.

**Reviewer:**

Adherence to ART is an important issue in the management of patients in ART programmes. I am surprised that the authors did not look at adherence while looking at key programme indicators. Although the authors indicate how monitoring of patients using viral loads was done before and after treat all, they did not look at this as one of their study outcomes. The authors need to acknowledge this as one of the weaknesses of this study.

**Response:**

Thank you very much. The before period included April-June 2015 and during this time VL had not been introduced for routine monitoring of ART response. Hence, we were not able to document the before-after effect of “Treat All” on VL.

**Reviewer:**

Specific comments:

Introduction:

In paragraph 2 of the introduction, the authors say that research in other countries demonstrated that after implementation of the “treat all”, people on ART had better outcomes. The authors should mention these countries and specifically mention which ART outcomes were better.

**Response:**

Thank you very much. We have revised the introduction section as per the advice by reviewer. The revised lines now read as follows

Studies from southern African countries have demonstrated the health and economic benefits of “Treat All”.  Research in rural South Africa and Malawi demonstrated that after implementation of “Treat All”, people on ART had better retention in care.

**Reviewer:**

The authors need to give more details on the 14% prevalence of HIV in Zimbabwe. The references given are for 2012, 2016 and 2018. Is the current HIV prevalence in Zimbabwe still 14%? How was this national prevalence of HIV estimated in Zimbabwe?

**Response:**

Thank you very much. The 2018 reference is to give the updated number of PLHIV in Zimbabwe (1.3 million). The 2016 reference is the ZIMPHIA survey (Zimbabwe Population-based HIV Impact Assessment) 2015-16 which gives the estimate for HIV prevalence among adults. The 2012 reference is the census and as it is not relevant, I have removed it from here (in the third para of introduction). The revised line now reads as follows

Zimbabwe has a generalized HIV epidemic, with an estimated 1.3 million PLHIV and an HIV prevalence of 14% among adults (15-49 years) as per the ZIMPHIA survey 2015-16.

**Reviewer:**

In the introduction, the authors should mention the proportion of HIV infected people who were taking ART before the treat all policy became operational and the proportion currently when treat all policy is operational.

**Response:**

Thank you very much. The trends in uptake of ART among PLHIV has been described by every quarter in the results section (see Figure 1). Hence to avoid duplication, we are not mentioning the same in the introduction. Also, comparative analysis of ART uptake is one of the objectives of the study. Hence, we are not mentioning it in the introduction. We hope this is fine.

**Reviewer:**

Methods:

Can you give more information on the 77 ART sites providing HIV diagnosis and treatment? Are these public facilities or private facilities?

**Response:**

Thank you very much. We have clarified in the revised manuscript that these are public facilities.

**Reviewer:**

Are there private providers of ART in Harare? If they are there, is the information captured by District health Information systems?

**Response:**

Thank you very much. Information on private providers is not captured in DHIS.

**Reviewer:**

Is data on ART adherence captured at these ART treatment sites?

**Response:**

Thank you very much. Data on ART adherence is collected but is unreliable as this is based on self-reports and pill count. Furthermore this is only available for those patients who returned for their last scheduled visit hence will not account for the LTFU and transfer-out patients.

**Reviewer:**

When people on ART get lost to follow up, is there anything done to establish reasons why they got lost to follow up?

**Response:**

Thank you very much. We have tried to speculate the reasons for this in the revised manuscript. An unpublished WHO report suggests that only 25% of those documented as LTFUs are true LTFUs. I am copy pasting the new lines added to the discussion section regarding this

High rates of LTFU before and during “Treat All” may not actually be LTFU but enrolment in a different clinic closer to home. Due to stigma and discrimination, people tend to get tested out of their area of residence where they are not known and after enrolment tend to move closer to home for treatment. Furthermore we have also added to the methods section in Paragraph 4 under the subheading “ ***ART initiation before “Treat All”***” that patients are declared LTFU if they are not successfully traced back to care within 90 days from their last scheduled visit either by phone call or physical follow-ups by community health workers.

**Reviewer:**

Discussion:

You need to acknowledge the weakness of not looking at adherence in this study. You actually seem to agree with me on this point where you say “another potential reason for the observed lower retention could be deficiencies in coverage of quality adherence counselling with likely increased work load during the “treat all era”.

**Response:**

Thank you very much. We agree with you. But as discussed before, data on ART adherence is collected but is unreliable as this is based on self-reports and pill count. Adherence would best be measured by VL testing which was not routinely available in the period prior to HIV treat All. Hence we were not in a position to compare the same before and during Treat All. Also there were large instances of missing data of CD4 count (mentioned as a limitation in second last paragraph of discussion)

**Reviewer:**

You also need to recommend qualitative studies to look at why retention remained low in the treat all era in Harare compared to other studies in other countries. You rightly said you speculated.

**Response:**

Thank you very much. We have included a line of qualitative systematic enquiry in discussion section.

The programme should consider qualitative systematic enquiry into why the retention did not improve during “Treat All” in Harare.

# Peer review: Reviewer response for version 1

**DOI:** 10.5256/f1000research.25846.r62743

**Published:** 2020-05-26

**Authors:** Boniface Dongmo‐Nguimfack

**Affiliations:** 1 Department of HIV/AIDS, World Health Organization, Geneva, Switzerland

- The study was designed to answer the question on uptake and retention comparing the before treat all period to the treat all period. Statistically speaking the analysis was well done and reflects the pure data analysis as well as the outcome of the analysis.
- The paper lacks a clear disclaimer of the limitation(s) of the study.
- No information on the lack of tracing one patient from one clinic to the other as soon as they get outside the ePMS.
- Lack of information on the mobility of patients in the city and the country in general.
- Lack of information on how easy or difficult it is to enroll in a new clinic.
- The lack of patient unique identifier outside the ePMS clinics.
- The difficulty when you reach the 80% enrolment to find the last 20%.
- No comparative analysis on how easy or difficult the enrolment and the treatment was within Harare between the before treat all period to the treat all period.
- The methodology does not reflect the reality of the country as there are still stigma and discrimination in the country and people tend to get tested out of their area of residence where they are not known and after enrollment tend to move closer to home for treatment which may justify the high rate of LTFU which actually may not be LTFU but enrollment in a different clinic closer to home.
- The WHO not-published survey in Harare in 2018 shows that out of 100 LTFU only 25 were LTFU, 75 have just changed clinics or regions.
- It is quite easy in Harare to move from one clinic to another clinic (the reason why the duplication in number is so high).
- WHO recommends index tracing and testing to move from 70% to 95% which is the new target.

Is the work clearly and accurately presented and does it cite the current literature?

Partly

If applicable, is the statistical analysis and its interpretation appropriate?

Yes

Are all the source data underlying the results available to ensure full reproducibility?

Yes

Is the study design appropriate and is the work technically sound?

Partly

Are the conclusions drawn adequately supported by the results?

Yes

Are sufficient details of methods and analysis provided to allow replication by others?

Yes

Reviewer Expertise:

I am an economist and member of the HIV guideline Technical Working Group of the WHO

I confirm that I have read this submission and believe that I have an appropriate level of expertise to confirm that it is of an acceptable scientific standard, however I have significant reservations, as outlined above.

# Response

**Published:** 2020-07-28

**Authors:** Hemant Deepak Shewade

**Affiliations:** International Union Against Tuberculosis and Lung Disease, India

Thank you very much for your comments. This has helped us improve our manuscript. We hope the revised manuscript now meets your expectations. Point by point response to your comments is provided below

**Reviewer:**

The study was designed to answer the question on uptake and retention comparing the before treat all period to the treat all period. Statistically speaking the analysis was well done and reflects the pure data analysis as well as the outcome of the analysis.

**Response:**

Thank you very much for the constructive comment.

**Reviewer:**

The paper lacks a clear disclaimer of the limitation(s) of the study.

**Response:**

Thank you very much. The strengths and limitations have been mentioned in the second last paragraph of discussion.

**Reviewer:**

No information on the lack of tracing one patient from one clinic to the other as soon as they get outside the ePMS.

**Response:**

Thank you very much. We agree with the reviewer here. We have added this as a limitation. We did not have details about what happened to those that were transferred out. Alive and on treatment and transferred out combined were combined as “retained in care”. Patients that were transferred out were censored on their last recorded visit to ART site.

**Reviewer:**

Lack of information on the mobility of patients in the city and the country in general.

**Response:**

Thank you very much. Yes, this is a limitation and is explained in second from last paragraph of the discussion section.

**Reviewer:**

Lack of information on how easy or difficult it is to enrol in a new clinic.

**Response:**

Thank you very much. We have added the following information under the methods subheading “ART initiation before “Treat All”:

If a patient officially transfers-out to another ART clinic, they are issued with a transfer-out letter for presentation at the receiving ART clinic and they maintain their ART number. In other instances, patients may unofficially transfer out to another health facility without being assigned a transfer-out letter and are therefore declared lost to follow-up at their original facility if they exceed 90 days after their last scheduled clinic or drug pick-up visit and all attempts to trace them back to care have been futile. The patients will often present at the transfer-in facility with a patient-held ART booklet which indicates their previous medical history and therefore continue receiving ART care whilst also maintaining their ART number. Alternatively, if no evidence of previous treatment is provided, they are offered an HIV test and subsequently assigned a new ART number and treated as new ART client

**Reviewer:**

The lack of patient unique identifier outside the ePMS clinics.

**Response:**

Thank you very much. We agree with the reviewer. However, we included only the ePMS sites which had electronic data for analysis. Hence, this was beyond the scope of our study. We also respond to your comment in the methods subheading “ART initiation before “Treat All””

**Reviewer:**

The difficulty when you reach the 80% enrolment to find the last 20%.

**Response:**

Thank you very much. We agree with the reviewer here based on our programme experience.

**Reviewer:**

No comparative analysis on how easy or difficult the enrolment and the treatment was within Harare between the before treat all period to the treat all period.

**Response:**

We have added some additional information in the methods section. Prior to “Treat All” psychosocial criteria for ART initiation, included completion of prescribed counselling sessions and an assessment of adherence to CPT in the past 3 months with CPT adherence being used to assess the likelihood that the patient would adhere to ART. Whereas in the Treat All era, additional changes included an emphasis for health workers to provide adequate counselling and start ART within a week with exception of pregnant and breast-feeding women who were to be started on ART on the same day of HIV diagnosis. However, for those patients who are not ready yet to start ART, they should receive on-going counselling and support.

**Reviewer:**

The methodology does not reflect the reality of the country as there are still stigma and discrimination in the country and people tend to get tested out of their area of residence where they are not known and after enrolment tend to move closer to home for treatment which may justify the high rate of LTFU which actually may not be LTFU but enrolment in a different clinic closer to home.

**Response:**

Thank you very much. We have included this point in paragraph four of the discussion section in the revised manuscript. We have copied it below for your kind perusal

High rates of LTFU before and during “Treat All” may not actually be LTFU but enrolment in a different clinic closer to home. Due to stigma and discrimination, people tend to get tested out of their area of residence where they are not known and after enrolment tend to move closer to home for treatment.

**Reviewer:**

The WHO not-published survey in Harare in 2018 shows that out of 100 LTFU only 25 were LTFU, 75 have just changed clinics or regions.

**Response:**

Thank you very much. This supports our revision in the fourth paragraph of discussion section.

**Reviewer:**

It is quite easy in Harare to move from one clinic to another clinic (the reason why the duplication in number is so high).

**Response:**

Thank you very much. We agree with the reviewer.

**Reviewer:**

WHO recommends index tracing and testing to move from 70% to 95% which is the new target.

**Response:**

Thank you very much. We agree with the reviewer. Index tracing and testing will help achieving the first (90) or the first (95) of the UNAIDS target. This deals with what proportion of expected HIV positive should be diagnosed. Our study dealt (objective on ART uptake) with the second 90 or 95 which states what percentage of diagnosed PLHIV should be started on ART. We hope this is fine.
